# Extraction of Various Magnesium Species from Slovenian Dolomites Using Chemical and Hydrometallurgical Approaches

**DOI:** 10.3390/ma19204300

**Published:** 2026-10-13

**Authors:** Jernej Imperl, Lea Žibret, Vilma Ducman, Gorazd Žibret, Neža Malenšek Andolšek, Zuzana Danková, Zuzana Kollová, Jaroslav Briančin, Mitja Kolar

**Affiliations:** 1 Faculty of Chemistry and Chemical Technology, University of Ljubljana, Večna Pot 113, 1000 Ljubljana, Slovenia; jernej.imperl@fkkt.uni-lj.si; 2 Slovenian National Building and Civil Engineering Institute, Dimičeva Ulica 12, 1000 Ljubljana, Slovenia; lea.zibret@zag.si (L.Ž.); vilma.ducman@zag.si (V.D.); 3 Geological Survey of Slovenia, Dimičeva Ulica 14, 1000 Ljubljana, Slovenia; gorazd.zibret@geo-zs.si (G.Ž.); neza.malensek@geo-zs.si (N.M.A.); 4 State Geological Institute of Dionýz Štúr, Regional Geological Service Košice, Jesenského 8, 040 01 Košice, Slovakia; zuzana.dankova@geology.sk (Z.D.); zuzana.kollova@geology.sk (Z.K.); 5 Institute of Geotechnics, Slovak Academy of Sciences, Watsonova 45, 040 01 Košice, Slovakia; briancin@saske.sk

**Keywords:** dolomite, magnesium, selective leaching, selective precipitation, magnesium hydroxide, calcium sulphate, calcination, thermal reduction

## Abstract

Although one of the most abundant elements, magnesium is classified as a critical raw material by the EU. Demand for Mg and its compounds is increasing because of their widespread applications and emerging potential uses in various industries. Most primary Mg metal production occurs outside the EU and is dominated by thermal reduction and molten salt electrolysis, both energy-intensive processes. Furthermore, converting dolomite, CaMg(CO_3_)_2_, one of the main Mg sources, into usable feed material requires high temperatures. In our study, the production of Mg(OH)_2_ was achieved through a two-step process involving selective leaching of Slovenian dolomite with H_2_SO_4_ and selective precipitation with hydroxide. The product contained 95% of the initial Mg and only 1% of Ca, resulting in a 97-fold enrichment in favour of Mg expressed as the Ca:Mg molar ratio. This process also supplied a CaSO_4_ by-product, a potential source of Ca. Modifying the precipitation step with the use of chelating agents EDTA, EGTA, and DTPA did not significantly affect the Mg yield, but reduced Ca traces, thereby increasing the molar ratio to over 200. As an alternative pathway, calcination and silicothermic reduction of the dolomite demonstrated its suitability for the production of crystalline metallic Mg.

## 1. Introduction

Magnesium (Mg) is one of the most abundant elements on Earth. It is widely distributed in the crust (2.1 wt.%) and oceans (0.1 wt.%) [1]. Magnesium is a versatile element whose various forms—metallic Mg, Mg alloys, and Mg compounds—have found many uses [2,3]. The metal itself is soft and highly reactive, but it has favourable electrochemical properties that make it particularly useful in chemical and industrial processing applications. In metallurgy Mg metal or Mg minerals are used as a reducing agent in the production of various other metals from their ores by thermal reduction [4] and as sacrificial anodes for corrosion inhibition [5]. The conversion of noncarbonate Mg minerals into Mg carbonate by way of carbonation is a novel approach to CO_2_ sequestration [6]. The low redox potential combined with the low weight gives Mg a higher volumetric capacity even compared with Li, the standard anodic material in batteries and energy storage devices, thus making Mg a promising substitute [7,8]. When combined with other metals, the Mg alloys produced are a lightweight alternative to many other metals and non-Mg alloys, while retaining comparable strength and other structural properties. Consequently, many transportation industries (automotive, railway, shipbuilding, aviation, and aerospace) have adopted Mg alloys to decrease the vehicle weight and thereby reduce the fuel consumption [9,10,11]. Other applications also include electronic devices, power tools, and sporting equipment [12,13]. Magnesium compounds used in medicine, agriculture, construction, and other manufacturing industries include the oxide MgO, the hydroxide Mg(OH)_2_, the sulphate MgSO_4_ × 7H_2_O, the carbonate MgCO_3_, and the chloride MgCl_2_ [14,15,16,17].

With the ever-increasing demand for magnesium and its products for the existing uses and the possibility of new potential applications, and the fact that all of the primary magnesium production occurs outside its borders (China representing 91% of total Mg production in the world and 97% of Eu-imports), the EU has classified magnesium as a critical raw material [18].

The most important sources of Mg are its carbonate ores (magnesite and dolomite), silicate ores (olivine and talc), evaporative chloride minerals (carnallite and bischofite), and the soluble Mg^2+^ cation in seawater [1,2]. Magnesium production mainly relies on two processes: thermal reduction and electrolysis. The usual feed material for thermal reduction is MgO, prepared from carbonate sources by calcination [19]. This preparation step requires heating magnesite (MgCO_3_) or dolomite (CaMg(CO_3_)_2_) to high temperatures (over 1000 °C) to expel CO_2_ and produce MgO or the mixed oxide CaO × MgO, known also as dolomitic lime [20]. MgO is then mixed with different reducing agents and heated in either a vacuum or an argon atmosphere to produce elemental Mg. When MgO is mixed with ferrosilicon (FeSi), the process is known as silicothermic reduction [21] and follows reaction (R1). Other types are alumothermic reduction, which uses powdered aluminium [22,23], and carbothermic reduction, which employs a source of carbon (e.g., coal) [24]. Reactions (R2) and (R3) illustrate these processes, respectively. In all cases, because of the high temperatures required (1100–1700 °C), the magnesium is produced as a gas and is then recondensed.

(R1) 2MgO + 2CaO + FeSi → 2Mg_(g)_ + Ca_2_SiO_4_ + Fe

(R2) 3MgO + 2Al → 3Mg_(g)_ + Al_2_O_3_

(R3) MgO + C → Mg_(g)_ + CO_(g)_

The calcination of dolomite ore followed by a silicothermic reduction of the resulting mixed oxide is more commonly referred to as the Pidgeon process and is responsible for the majority of primary Mg production worldwide [25,26]. Various improvements of the thermal reduction process have been explored, including microwave heating to improve heat transfer and reduce energy consumption [27], performing the reduction at a normal pressure instead of the usually required vacuum or reduced Ar atmosphere [23], and modifying the pellet structure to make it more porous to improve the reduction efficiency and increase the Mg yield [28].

The other option is molten salt electrolysis. The industrial electrolytic process involves the use of anhydrous MgCl_2_, which is produced either from chloride minerals, by dehydration and purification of brine, or by conversion of other Mg ores or resources through leaching with HCl [1,29,30,31,32]. The feed MgCl_2_ material is melted and electrolysed to produce liquid Mg metal at the cathode and chlorine gas at the anode [33]. The Mg is then removed and isolated. Because anhydrous MgCl_2_ is difficult to produce and requires a lot of energy for dehydration, and, for the additional reason that toxic chlorine gas is produced during electrolysis [34], novel approaches have been researched and developed that use MgO, a product of calcination of Mg carbonate ores, as feedstock for the electrolytic process. This is combined with electrodes made from different materials to improve the energy efficiency, yield, and purity of the produced Mg [33,35,36,37].

In recent years, various hydrometallurgical methods have been explored and developed to replace or complement the aforementioned routes, as they offer solution-based pathways to Mg products that are less energy-intensive and operate at low temperatures [38]. Source materials are usually partially or totally dissolved in a reagent solution (typically an acid), forming a Mg-rich solution that can be processed further (precipitation, purification, or synthesis of Mg compounds) [39]. Mg can be selectively precipitated from brine as the hydroxide Mg(OH)_2_, as it is much more insoluble than other common metal ions present in brines [40,41]. Solid Mg sources, such as its silicate and carbonate ores, can be dissolved to release Mg ions. While silicates are more stable and resistant to attack by acid [42,43,44] or base [45] and therefore require harsher conditions, carbonates are readily dissolved in different inorganic or even organic acids. The use of HCl (total dissolution, 98.82% of Mg extracted from calcined dolomite) [46], HNO_3_ (total dissolution) [47], H_2_SO_4_ (partial dissolution, Ca precipitation yield of 93.37%) [48], and H_3_PO_4_ (partial dissolution, leaching yield 16.64% of Ca and 93.44% of Mg) [49] for total or partial dissolution has already been reported. The latter two can also aid the separation of Ca and Mg, as the sulphates and phosphates of these metals have large differences in solubility. Among the organic acids, gluconic acid (calcined magnesite, close to 100% Mg extracted) [50], citric acid (calcined magnesite, 98.86% of Mg extracted) [51], acetic acid (calcined magnesite, approx. 80% of Mg extracted) [52], succinic acid (magnesite, 88% of Mg extracted) [53], and formic acid (magnesite, 95% of Mg extracted) [54] have been used. Although organic acids have limited dissolution power, some are highly selective and can separate Ca and Mg ions present in dolomite [39]. Despite promising extraction yields achieved with mineral acids, organic acids, reactive mixtures, and sequential leaching–precipitation processes in the hydrometallurgical field, their commercial viability is hampered by the fragmented methodologies and poor operational reporting, as many operational parameters influence the outcome of such processes [38,39].

**Figure 1:**
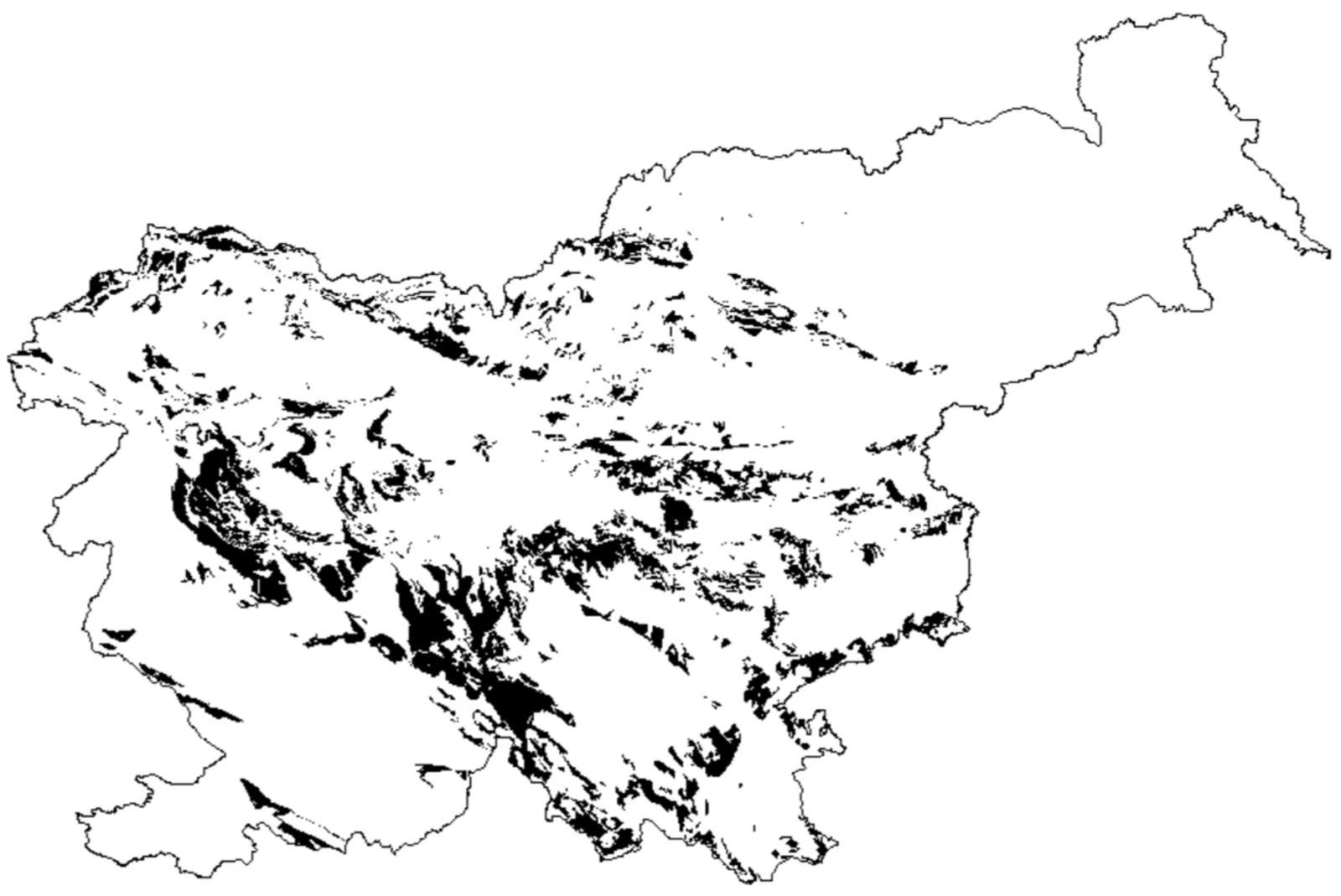
Dolomite areas in Slovenia [60].

Dolomite is a carbonate mineral of Mg and Ca with the empirical formula CaMg(CO_3_)_2_, from which it follows that it contains equal amounts of both metals (the molar ratio is 1:1), or, expressed in mass percentages, it contains 13.18 wt.% Mg. Dolomite can also refer to the type of rock, where its major component is the mineral dolomite, while limestone is composed mainly from the mineral calcite, CaCO_3_. The territory of the Republic of Slovenia is predominantly (more than 90%) composed of sediments and sedimentary rocks [55], of which carbonates cover around 40% of the surface. Limestone and dolomite are the principal types of carbonates, with the latter forming 12.4% of Slovenia’s surface (Figure 1) [56,57].

Dolomite is predominantly formed through a geological process known as dolomitisation, in which Ca ions in pure Ca minerals, such as calcite, are exchanged with Mg ions [58]. This occurs when the Ca-containing mineral or rock is in contact with a Mg-containing solution. Only rarely did the dolomite form directly by precipitation [19]. The formation of dolomite by dolomitisation in Slovenia occurred across different geological periods—from the Permian to the Cretaceous [55,59], with the majority formed in the Permian and Triassic periods [60,61].

The EU’s policies aim to increase self-sufficiency and its designation of Mg as a critical raw material, together with the anticipated increased demand for various forms of magnesium, prompted us to carry out the present research. We aimed to investigate the use of Slovenian dolomites in a broader sense, concentrating high-Mg fractions using simple methods and affordable reagents. Additionally, we sought to determine whether the abundant domestic dolomites can be used as a source for direct Mg preparation.

## 2. Materials and Methods

### 2.1. Dolomite Samples Selection

Of the 48 dolomite samples collected across Slovenia as part of an ongoing project, five samples were selected for further use in testing the potential of Mg extraction. The samples were chosen according to several criteria: all were geologically identified as pure dolomite, they were collected from major deposits, and they represented all geological periods in which dolomitisation is known to have occurred (Table 1) [55,60]. The samples were collected in bulk and ground into a fine powder using Fritsch Pulverisette W-carbide jaw and disc crushers with final fineness of 0.05 mm. The powdered samples were stored in airtight transparent plastic containers.

### 2.2. Elemental Analysis

The elemental composition of selected samples was determined by total microwave-assisted acid digestion. Approx. 250 mg of a dolomite sample was weighed into a PTFE vessel, and 8 mL of concentrated HCl (37%, p.a., KEFO, Ljubljana, Slovenia) and 2 mL of HBF_4_ solution were added. The HBF_4_ solution was prepared fresh in the laboratory by dissolving H_3_BO_3_ (p.a., Honeywell, Charlotte, NC, USA) in concentrated HF (48%, p.a., Merck, Darmstadt, Germany) in a ratio of 0.4 g of H_3_BO_3_ to 1 mL of HF. The total acid digestion was carried out in a microwave system (Ethos UP, Milestone, Sorisole, Italy) using a three-step temperature programme: 1. heating to 220 °C over 30 min, 2. maintaining the set temperature (220 °C) for 15 min, and 3. cooling to near room temperature. After opening, the sample solution was quantitatively transferred into a 50 mL graduated centrifuge tube (polypropylene, Sarstedt, Nümbrecht, Germany) and made up to the mark with ultrapure water (resistivity >18.2 MΩ cm, Synergy Water Purification System, Merck Millipore, Darmstadt, Germany). The samples were digested in six replicates. The blank sample was prepared in the same way.

### 2.3. Selective Leaching

Selective leaching of Mg was carried out using H_2_SO_4_ (96%, p.a., Honeywell, Charlotte, NC, USA). The dolomite sample was accurately weighed (1.00 ± 0.01 g) into a 100 mL Erlenmeyer flask, and 5.0, 7.5, 10.0, or 20.0 mL of H_2_SO_4_ solution was added with a pipette. Each H_2_SO_4_ solution was prepared in such a way that the volume added to the dolomite sample contained a certain percentage (50%, 105%, or 150%) of the stoichiometric amount required for complete reaction with the combined amounts of Mg and Ca in 1 g of the dolomite sample (as determined by elemental analysis). The flask was then shaken on an orbital shaker (Labnet, Edison, NJ, USA) at 180 rpm for 1 h at room temperature. After shaking, the mixture was filtered through filter paper (white mark, IDL, Nidderau, Germany) into a 50 mL centrifuge tube. The flask and solid residue on the filter were washed with two 5 mL portions of reagent solution diluted 1000-fold with ultrapure water. The filtrate was acidified by adding 1 mL of concentrated HNO_3_ and made up to the mark with ultrapure water. The solid residue was dried on the filter paper to constant mass (3 h at 110 °C) in a drying oven (Kambič, Semič, Slovenia), weighed, and stored in a 50 mL centrifuge tube. The samples were leached in six replicates.

### 2.4. Selective Precipitation

Selective precipitation of Mg was carried out using NaOH (p.a., Merck, Darmstadt, Germany). In the first step, the dolomite sample was accurately weighed (1.00 ± 0.01 g) into a pre-weighed 50 mL centrifuge tube and dissolved in 10 mL of HNO_3_ solution prepared to contain 105% of the stoichiometric amount of the total amount of Mg and Ca present in 1 g of the dolomite sample (as determined by elemental analysis). Dissolution was aided by placing the centrifuge tube in a sonication bath (Iskra, Ljubljana, Slovenia). In the second step, 10 or 20 mL of NaOH solution was added with a pipette to the dissolved dolomite solution. The NaOH solution was prepared to contain 105% of the stoichiometric amount of only total Mg present in 1 g of the dolomite sample (as determined by elemental analysis). The centrifuge tube was then closed and shaken horizontally on the orbital shaker at 210 rpm for 1 h. The resulting mixture was centrifuged at 4000 rpm for 5 min (Domel, Železniki, Slovenia) at room temperature. The supernatant was decanted into a new 50 mL centrifuge tube and stored. To the residue, 5 mL of NaOH solution diluted 1000-fold was added, vigorously shaken by hand, and centrifuged again. This was repeated twice. The new supernatants were combined in a separate 50 mL centrifuge tube and stored. The residue was dried in the centrifuge tube to constant mass (3 h at 110 °C) in the drying oven, weighed, and stored. Lastly, the dried residue was calcined at 950 °C for 1 h in a furnace (Snol, Utena, Lithuania). The samples were prepared in six replicates.

### 2.5. Combination and Modification of the Separation Procedures

In further experiments, the resulting filtrate from the selective leaching process with H_2_SO_4_ (10 mL with 105% stoichiometric amount) was stored and used for selective precipitation with NaOH, thereby combining the processes.

In additional experiments, the precipitation step was modified with the use of 3 chelating agents. The NaOH solution, used for selective precipitation in the second step of the combined procedure, was prepared with three chelating agents: EDTA (Na_2_EDTA × 2H_2_O, p.a., Gram-Mol, Zagreb, Croatia), EGTA (p.a., Fluka, Honeywell, Charlotte, NC, USA), and DTPA (p.a., Fluka, Honeywell, Charlotte, NC, USA). The solution contained the same amount of NaOH as before and a chelating agent amounting to 1% of the stoichiometric amount of total Ca present in 1 g of the dolomite sample (as determined by elemental analysis).

### 2.6. Calcination and Silicothermic Reduction

Calcination and silicothermic reduction was done under laboratory conditions according to the procedure described by Danková et al. [62,63]. A sample of dolomite was first calcined in a high-temperature muffle furnace (KSL-1700X-A2, MTI Corporation, Richmond, CA, USA) at 1200 °C for 1.5 h (heating step at 10 °C/min) to prepare the mixed oxide CaO × MgO (dolomitic lime). In the second step, it was mixed with FeSi and CaF_2_ in a ratio of 77.5% CaO × MgO, 17.5% FeSi, and 5.0% CaF_2_. The resulting mixture was then pressed into pellets weighing approximately 1 g each. Three pellets were loaded into an in-lab-constructed horizontal furnace and fired in an argon atmosphere at 1150 °C for 4 h (heating step at 4 °C/min) to reduce MgO to elemental Mg. A copper tube with flowing cold water passed through the furnace to ensure the isolation and collection of evaporated Mg. Metallic Mg product was stored in a desiccator under Ar.

### 2.7. Characterisation

Sample solutions and solid products were analysed by inductively coupled plasma optical emission spectrometry (ICP-OES). The solid products were accurately weighed and redissolved in concentrated HNO_3_. All sample solutions were appropriately diluted with 1% HNO_3_ solution, prepared from concentrated HNO_3_ and ultrapure water, and measured with inductively coupled plasma optical emission spectrometer (ICP-OES, Agilent 5100 SVDV, Agilent Technologies, Santa Clara, CA, USA). Quantification was performed using a nine-point linear calibration curve ranging from 0.1 to 1000 µg/L (R^2^ > 0.999). Standards were prepared by diluting two certified multi-element standard solutions—Periodic Table Mix 1 for ICP (33 elements, 10 mg/L, Merck, Darmstadt, Germany), and VAR-CAL-1 (4 elements, 100 mg/L, Inorganic Ventures, Christiansburg, VA, USA) with 1% HNO_3_ solution. A total of 36 elements were determined: Li, Be, Na, Mg, Al, Si, P, S, K, Ca, V, Cr, Mn, Fe, Co, Ni, Cu, Zn, Ga, As, Se, Rb, Sr, Ag, Cd, In, Te, Ba, Tl, Pb, Bi, and Ti, Mo, Sn, Sb.

Dolomite samples and solid products were additionally analysed by scanning electron microscopy (SEM), electron-dispersive X-ray spectroscopy (EDS), thermogravimetric analysis (TGA), and X-ray diffraction (XRD).

Morphological characterisation was carried out using a high-resolution field-emission scanning electron microscope (FE-SEM, Thermo Fisher Scientific, Waltham, MA, USA). Powder samples were mounted onto conductive carbon tape (Agar Scientific, Rotherham, UK) attached to aluminium SEM stubs. To prevent surface charging during imaging, non-conductive samples were sputter-coated with a 20 nm Au/Pd (80:20) layer prior to imaging. SEM images were recorded at an accelerating voltage of 2 kV and a beam current of 13 µA using ETD side-mounted SE2 and T1 segmented in-lens detectors at different working distances.

Elemental composition was evaluated using an energy-dispersive X-ray spectrometer (EDS, Oxford Instruments, Abingdon, UK) equipped with an Oxford Instruments Ultim Max 100 detector. EDS measurements were conducted at an accelerating voltage of 10 kV and a beam current of 0.2 nA. Spectrum processing and elemental mapping were completed using the Aztec software package (Suite 6.3).

Thermal decomposition behavior of dolomite samples was evaluated using a TGA/DSC1 instrument (Mettler Toledo, Greifensee, Switzerland). Approximately 10 mg of sample was placed in a crucible and loaded into the instrument (TGA/DSC1, Mettler Toledo, Greifensee, Switzerland). The measurement was performed under a high-purity air and Ar atmosphere with a flow rate of 100 mL/min. The temperature programme comprised an isothermal step holding 25 °C for 15 min and a heating step increasing the temperature to 1000 °C at the rate of 5 K/min.

Mineralogical phase analysis of the precipitates was performed on a PANalytical Empyrean X-ray diffractometer utilising CuKα radiation (wavelength CuKα1 1.54 Å). Samples were ground in an agate mortar to a particle size below 63 μm and manually back-loaded into a 27 mm diameter cylindrical sample holder. XRD patterns were recorded over a 2*θ* range of 4° to 70° with a step size of 0.013° while continuously rotating the sample stage. The X-ray tube was operated at 45 kV and 40 mA. Phase identification was conducted using PANalytical X’Pert HighScore Plus software (v. 4.8) matched against the ICDD PDF-4+ 2016 RDB database.

## 3. Results and Discussion

### 3.1. Elemental Analysis

Elemental analysis confirmed the geological identification of the selected samples as pure dolomite. Apart from DOL43, all samples had an almost ideal composition with respect to the expected major components Ca and Mg, expressed as mass percentages and calculated molar ratios of both metals (Table 2). All samples contained more Ca and less Mg than the ideal values of 21.74 wt.% and 13.18 wt.%, respectively. Thus, the calculated molar ratios of the dolomite samples leaned slightly towards Ca. The ideal values were calculated from the chemical formula of the mineral dolomite, CaMg(CO_3_)_2_.

DOL43 was the youngest sample (Cretaceous) and exhibited an incomplete dolomitisation process, as the mass percentages of Ca and Mg were furthest apart at 24.05 wt.% and 11.69 wt.%, respectively, and the Ca:Mg molar ratio was the lowest at 0.802. In terms of purity, the DOL1 sample contained the smallest amount of elemental impurities, totalling less than 0.1 wt.%, which was consistent with its appearance as a white powder. It was also the only sample where the main impurity was not Si but rather Zn. The other samples contained more impurities, with the highest being 0.6 wt.% in DOL37. The impurities present in all samples consisted of common mineral elements such as Na, Al, Si, K, Fe, and Zn (Table S1). To confirm the elemental composition and purity of sample DOL1, SEM-EDS and TGA analysis were performed. SEM-EDS showed an almost ideal distribution of elements Ca, Mg, C, and O by mass percentages (Figure S1) and confirmed the results of the elemental analysis with ICP-OES. TGA determined that the LOI was the same as would be expected for the thermal degradation of ideal dolomite, where the loss of mass corresponds to the loss of CO_2_ gas [62]. The LOI of DOL1 in either air (47.38 wt.%) or argon (47.31 wt.%) closely matches the theoretical value of 47.73 wt.% (Figure S2).

DOL1 was chosen as the model dolomite for testing Mg extraction using selective leaching and selective precipitation. The decision was based on the results of the elemental analysis and other practical considerations, such as the sample DOL1 came from the largest deposit and was readily accessible in greater quantities. Although this sample contained lower amounts of both Mg and Ca than the other samples, the molar ratio was practically identical. Furthermore, it contained very few major impurities, and in smaller amounts, specifically Si, which would cause the sample not to be completely dissolvable in common mineral acids such as H_2_SO_4_ and HNO_3_.

### 3.2. Selective Leaching

The separation of Mg and Ca is difficult, because the elements are chemically very similar and behave and react in analogous ways. Nevertheless, there are some differences that can be exploited to separate the two metals. One of these is the solubility, or rather insolubility, of Mg and Ca compounds with certain reagents. Dissolving dolomite with H_2_SO_4_ results in the formation of magnesium sulphate, MgSO_4_, and calcium sulphate, CaSO_4_, with the former being much more soluble than the latter. MgSO_4_, whose hydrated form is commonly known as Epsom salt, is highly soluble and is more than a million times more soluble than its calcium analogue (K_sp_ = 3.2 × 10^−5^ as CaSO_4_ at 25 °C) [64]. Therefore, the dissolution of dolomite with H_2_SO_4_ should produce a solid precipitate of CaSO_4_ and a Mg-rich solution, thereby selectively dissolving or leaching only the Mg.

To test this with the model dolomite, we used different volumes of H_2_SO_4_ solution containing 50%, 105%, or 150% of the stoichiometric amount (combined amount of Ca and Mg in 1 g of dolomite DOL1) to selectively leach Mg (Table 3). The results are expressed as elemental yield calculated by Equation (1):

(1) $$\mathrm{elemental yield}\;\eta \left[\%\right]=100\times \frac{m_{\mathrm{element}-\mathrm{determined}}}{m_{\mathrm{element}-\mathrm{dolomite}}}$$

where *m*_element-determined_ stands for the mass of the element determined in the resulting solution and *m*_element-dolomite_ stands for the mass of the element in the dolomite sample (calculated from the mass of each dolomite sample times the mass percentage of the element in DOL1 as determined by elemental analysis).

The reaction between dolomite and H_2_SO_4_ (R4) produces four products—dissolved Mg ions in the form of MgSO_4_, precipitate of CaSO_4_, liquid water, and CO_2_ as gas.

(R4) $$\mathrm{CaMg}{\left({\mathrm{CO}}_{3}\right)}_{2}+{2H}_{2}{\mathrm{SO}}_{4}\to {\mathrm{MgSO}}_{4(\mathrm{aq})}+{\mathrm{CaSO}}_{4(s)}+{2H}_{2}O_{\left(l\right)}+2CO_{2\;\left(g\right)}$$

The results from leaching with only a 50% stoichiometric amount show that H_2_SO_4_ has no preference for reacting with either Ca or Mg, as the Mg yield was marginally below the expected 50%. With 100% and 150% stoichiometric amount of H_2_SO_4_, the dissolution of dolomite was complete, as almost all of Mg was dissolved. Leaching with a 150% stoichiometric amount did not improve Mg leaching efficiency in either experiment using 10 or 20 mL of H_2_SO_4_ solution. While the Mg yield even decreased slightly compared with the 105% stoichiometric amount, more Ca was extracted, with the yield increasing by 1–2 percentage points. Using volumes smaller than 10 mL presented a practical hurdle, as the resulting suspension did not mix well when shaken and the filtration was hindered. This is evident from the lower Mg yield (86.7%) when only 5 mL of H_2_SO_4_ solution was used. Using 7.5 mL of H_2_SO_4_ solution did give a higher Mg yield than in the best case, but the margin of error was greater, and more Ca was extracted as well, lowering the Ca:Mg molar ratio from 19.1 to 17.5. A larger volume (20 mL) had no effect on the Mg extraction efficiency but caused more of the CaSO_4_ precipitate to be dissolved, increasing the Ca yield and decreasing the molar ratio. In the best case, where Mg was leached from the dolomite using 10 mL of H_2_SO_4_ solution with a 105% stoichiometric amount, we obtained a solution in which more than 95% of the total Mg and only 5% of the total Ca were dissolved. Expressed as a Ca:Mg molar ratio, the solution contained 19 times more Mg than Ca.

### 3.3. Selective Precipitation

In addition to metallic magnesium, Mg compounds (oxide, hydroxide, and chloride) are industrially important forms of Mg as previously mentioned in the literature [11,15,16]. For this reason, we investigated precipitating Mg as magnesium hydroxide, Mg(OH)_2_. Although the hydroxides of both metals are insoluble, the difference in their solubilities is extremely large; in this case, calcium hydroxide, Ca(OH)_2_ (pK_sp_ = 5.3 at 25 °C), is almost a million times more soluble than Mg(OH)_2_ (pK_sp_ = 10.8 at 25 °C) [65]. Consequently, it is possible to selectively precipitate only Mg(OH)_2_ from a solution containing both Ca and Mg ions.

To investigate if this is possible with the model dolomite, DOL1 was dissolved in HNO_3_ solution, prepared to contain 105% of the stoichiometric amount (combined amount of Ca and Mg in 1 g of dolomite DOL1). The sample was completely dissolved, producing a concentrated solution of both elements. This was confirmed by preparing additional samples and analysing them with ICP-OES (>98% yield for Ca and Mg). Different volumes of NaOH solution, containing 105% of the stoichiometric amount of only the total Mg in 1 g of dolomite sample DOL1, were then added. There was no discernible difference between using 10 or 20 mL of the NaOH solution. In both cases, the majority of Mg (>90%) precipitated as Mg(OH)_2_ according to reaction (R5) and most of the Ca (approx. 95%) remained in the solution (Table 4). The solid product was about 15 times richer in Mg than the starting dolomite DOL1. The smaller volume of NaOH was chosen as the optimal precipitation step.

(R5) Mg^2+^+2OH^−^→Mg(OH)_2 (s)_

### 3.4. Combination of Selective Leaching and Selective Precipitation

**Figure 2:**
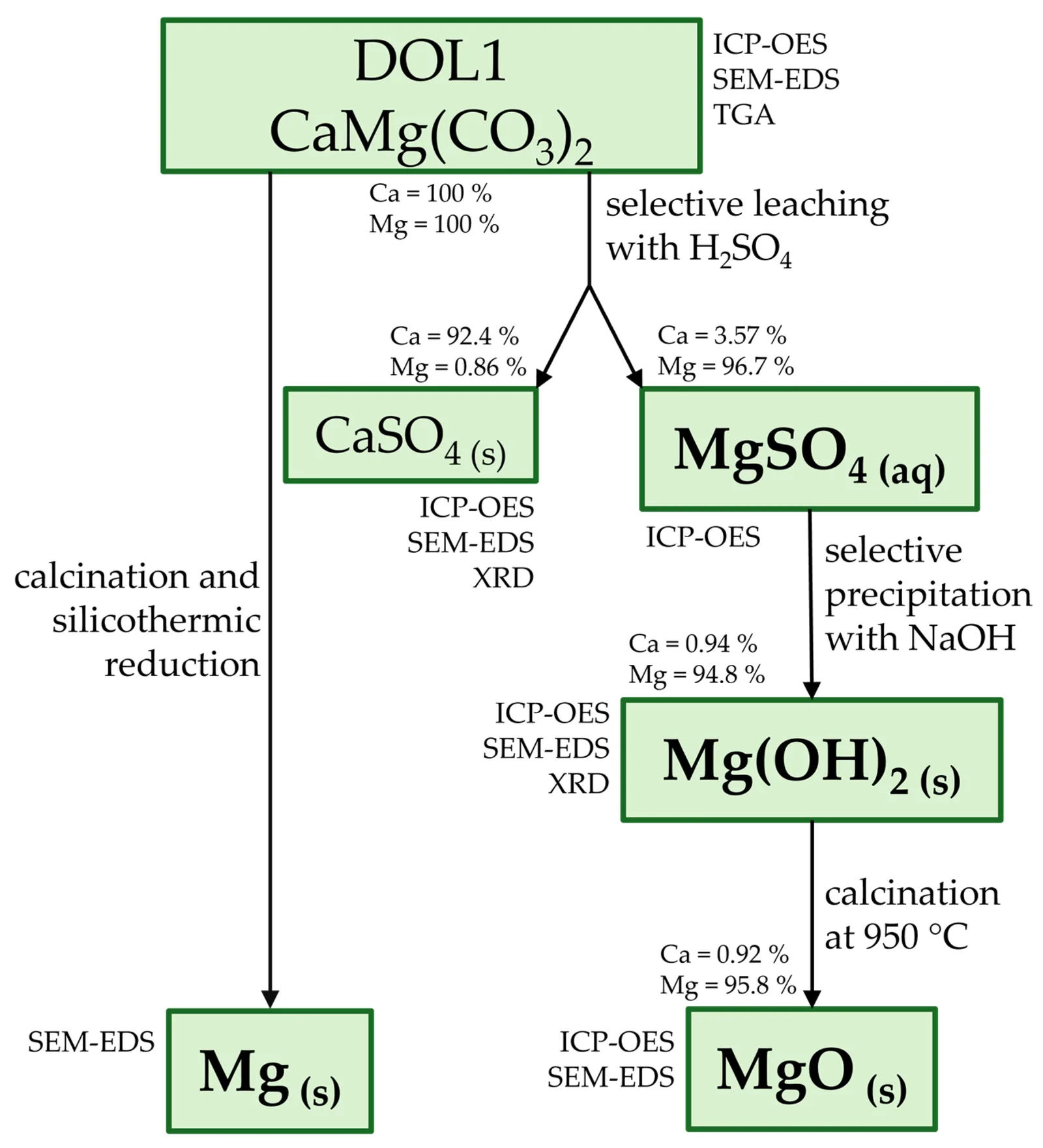
Extraction and production of Mg compounds from DOL1, accompanying analyses, and mass yields in each step.

We also investigated combining selective leaching and selective precipitation in a sequential two-step procedure. The MgSO_4_ solution produced under optimised selective leaching conditions was used as the starting solution for selective precipitation to produce Mg(OH)_2_. In the final stage, the precipitate was calcined and we obtained the end product, magnesium oxide (MgO). The workflow, the analyses performed, and the mass yields in each step are schematically illustrated in Figure 2.

The selective leaching of DOL1 with H_2_SO_4_ was successfully repeated under identical conditions and even gave slightly improved results. The Ca:Mg molar ratio of the produced MgSO_4_ solution increased to 26.0, as the average Mg yield rose (from 95.6% to 96.7%) and the Ca yield fell (from 4.81% to 3.57%) (Table 5). At the same time, the precipitated CaSO_4_ was collected, dried, and analysed. It was found to contain more than 92% of the total Ca and less than 1% of the total Mg, thus constituting a very rich Ca phase; the Ca:Mg molar ratio of 0.009 is heavily skewed towards Ca. Both phases together contained 96% of the starting Ca and almost 98% of the starting Mg, showing that the dolomite DOL1 was completely dissolved by H_2_SO_4_.

**Figure 3:**
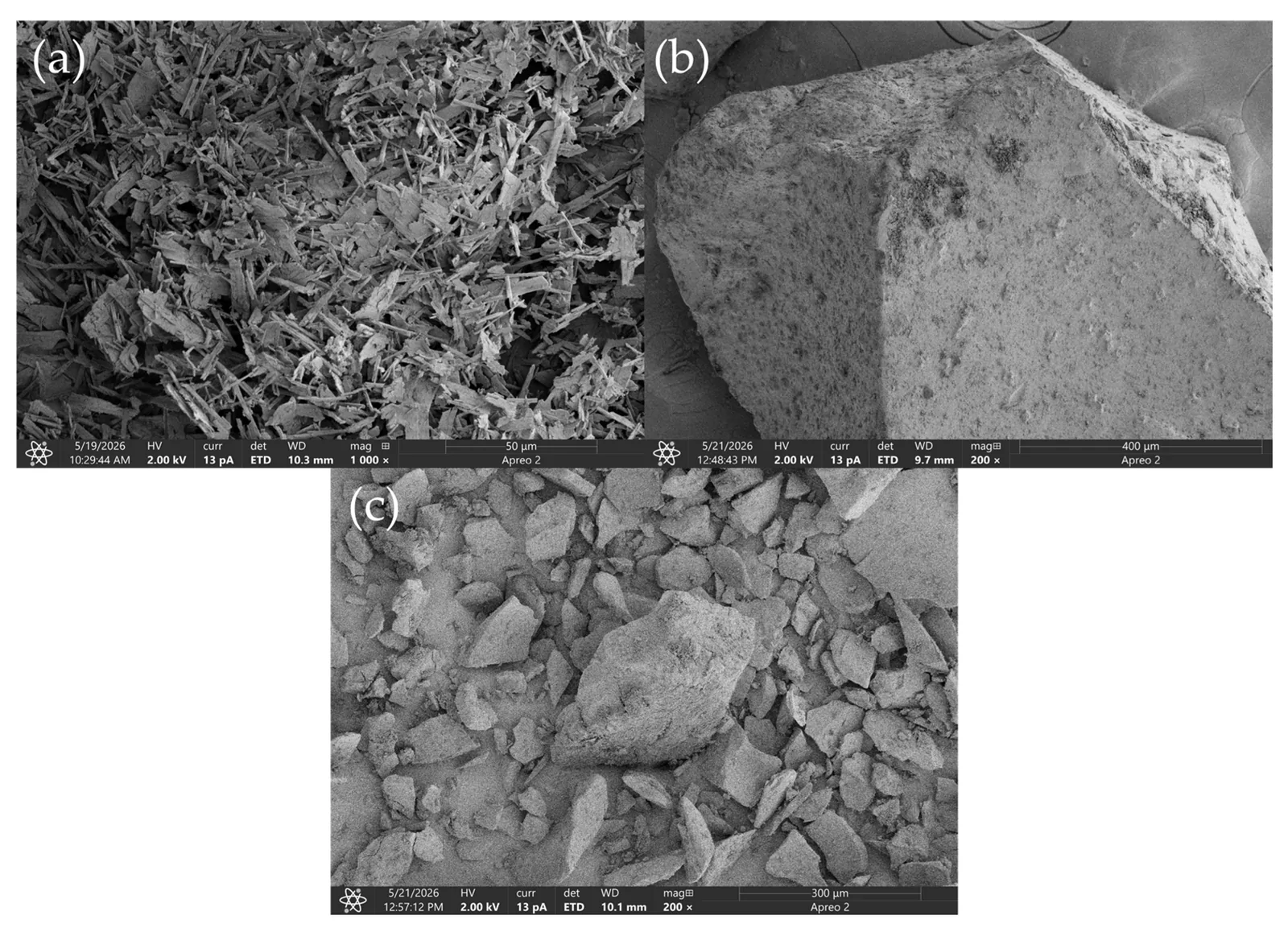
SEM images of (**a**) CaSO_4_, (**b**) Mg(OH)_2_, and (**c**) MgO.

SEM imaging of the solid CaSO_4_ showed needle-like crystals (Figure 3a), consistent with the crystal morphology of anhydrite (CaSO_4_) or gypsum (CaSO_4_ × 2H_2_O) [66]. EDS of the solid CaSO_4_ confirmed the elemental composition determined with ICP-OES (Figure S3a). XRD analysis was also performed and the obtained CaSO_4_ phase was identified as hemihydrate (CaSO_4_ × 0.5H_2_O) (Figure S4). Thus, the efficient separation of the two metals was achieved. A Mg-rich solution and solid CaSO_4_ were obtained, with the latter formed as a by-product but representing a potential material resource itself [67,68,69] or a source of calcium and its other compounds [70,71].

In the second step, Mg was precipitated by adding NaOH solution to the MgSO_4_ solution from the first step. The precipitated Mg(OH)_2_ was stored, dried, and analysed. It was determined that almost 95% of the initial Mg from the dolomite precipitated, and that the precipitate was a Mg-rich Mg(OH)_2_, as the Ca yield decreased to below 1% compared with the 3.57% found in the MgSO_4_ solution (Table 5). Precipitation of Mg from the solution was complete, comparing the 96.7% of total Mg dissolved from dolomite and 94.8% of total Mg precipitated means that 98% of the dissolved Mg precipitated. As NaOH was used, the solid product did contain some sodium impurities (2.00 wt.%) even after all the washing steps. This was also confirmed by XRD analysis (Figure S5), which identified brucite Mg(OH)_2_ as the main phase with the spectrum also containing trace signals of thenardite (Na_2_SO_4_), which is consistent with the chemicals used. The elemental composition was again confirmed by performing an EDS analysis of the solid Mg(OH)_2_ (Figure S3b), and the sample was also imaged with SEM (Figure 3b). The purification was confirmed by analysing the solution left after precipitation and washing, which contained most of the remaining traces of Ca (2.34% yield) and almost no Mg (0.38% yield). Combining both procedures was therefore efficient, as the yields of both metals in the Ca by-product and the Mg main product were over 90%, and it afforded the production of a source of Mg as Mg(OH)_2_—the Ca:Mg molar ratio stood at 96.7.

Ultimately, the Mg(OH)_2_ was calcined at 950 °C to obtain the final product, magnesium oxide MgO, practically without any losses. The Ca and Mg yields, and the Ca:Mg molar ratio are essentially the same as the values for Mg(OH)_2_ (Table 5). The morphology of the solid MgO was studied with SEM (Figure 3c). The elemental composition determined by ICP-OES was checked with EDS (Figure S3c) and confirmed the end product was indeed MgO.

### 3.5. Modification of the Separation Procedure

To further purify the produced Mg(OH)_2_ and, by extension, the final MgO product, the selective precipitation step was modified by using the chelating agents EDTA, EGTA, and DTPA. These were added to the NaOH solution to suppress the coprecipitation of the residual Ca present in the MgSO_4_ solution, because all form more stable Ca complexes than Mg complexes (Table S2) [72]. The amount of 1% of the stoichiometric amount of the total Ca was chosen based on the results, that the final Ca yield in the Mg(OH)_2_, produced in the two-step procedure, was just under 1%. The prediction of lowering the final Ca yield by the formation of soluble Ca-complexes that made Ca unavailable for precipitation with hydroxide was supported by the elemental analysis results for solid Mg(OH)_2_ (Table 6).

Although the average Mg yield was somewhat lower by 2–3 percentage points than in the case without any chelating agent, the average Ca yield was significantly lower by a factor of 2–3 (reduced from 0.94% to 0.33–0.43%). This led to a large increase in the Ca:Mg molar ratio, which nearly tripled from 96.7 to 269 in the best case involving the use of EGTA. The reduction of Ca traces can be explained by the formation of stable soluble Ca-complexes that made Ca ions unavailable for precipitation with hydroxide. The lower Mg yield also indicates that some Mg-complexes were formed as well. Considering the results of the analysis of the solid Mg(OH)_2_ product and, in addition, the results of the analysis of the solution after precipitation, it can be observed that the efficiency of Mg extraction followed the differences between the complex formation constants (Table S2). EGTA, which has the largest difference in favour of the formation of the Ca–EGTA complex, gave the best results in terms of the Ca:Mg molar ratio in the solid product and the highest Ca yield, as well as the lowest Mg yield (lowest Ca:Mg molar ratio) in the remaining solution after precipitation.

### 3.6. Calcination and Silicothermic Reduction

**Figure 4:**
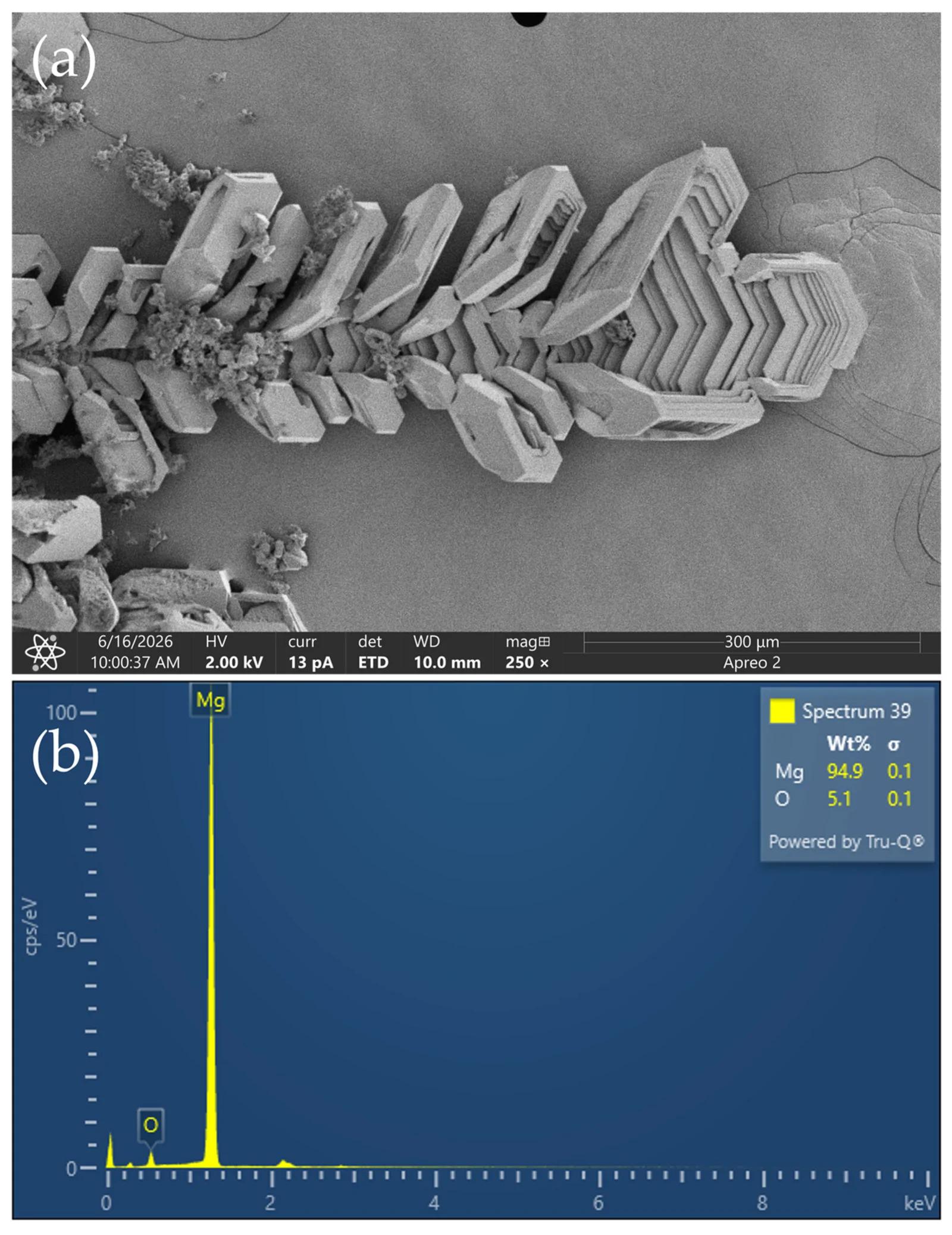
(**a**) SEM image and (**b**) EDS analysis of crystalline metallic Mg.

As an alternative and a well-established pathway of Mg production, the dolomite sample DOL1 was also used for the direct production of metallic Mg through calcination and silicothermic reduction according to the laboratory procedure [62,63]. DOL1 was chosen because of its ideal composition and low impurity content, as high quality and purity are preferable to minimise side reactions and avoid the formation of mixed metal fractions. The procedures were successfully used to produce metallic Mg on a laboratory scale. The formation of deposited metallic Mg crystals was confirmed by observing them using SEM imaging (Figure 4a). EDS (Figure 4b) analysis showed that the isolated Mg sample contained 95 wt.% Mg and trace amounts of oxygen from the oxidation of the Mg metal.

## 4. Conclusions

The presented study demonstrates that the hydrometallurgical processes of dissolution and precipitation of solid dolomite, one of the main sources of Mg, can produce separate Ca- and Mg-rich fractions. Although both alkaline earth elements share near-identical ionic properties and reactivity, their behaviour under competitive sulphate dissolution and hydroxide precipitation differs sufficiently to enable their partition without exotic reagents.

The first H_2_SO_4_ acid leaching stage exploited the large differences in sulphate solubility, with MgSO_4_ being the more soluble of the two. Total dissolution of dolomite with H_2_SO_4_ produced a Mg-rich solution containing almost 97% of the initial Mg in the dolomite and a solid CaSO_4_ precipitate as a by-product, capturing 92% of the initial Ca. Subsequent NaOH alkaline treatment of the Mg-rich solution reversed the solubility preference. This time, Mg precipitated as a hydroxide due to its lower solubility product, while trace Ca ions remained soluble. Combining these two distinct separation mechanisms into a two-step sequence created a synergistic effect that resulted in the formation of the main product, Mg(OH)_2_, containing almost 95% of the initial Mg and only trace amounts of Ca (less than 1% of the initial Ca), giving a high enrichment factor of 96.7, expressed as the Ca:Mg molar ratio. This provides a simple and efficient route to produce valuable Mg products using cheap, industrially produced and readily available chemicals together with straightforward procedures.

Furthermore, introducing low-concentration chelating agents led to competitive complex formation that preferentially sequestered trace Ca ions. Although less Mg was extracted, the decrease in the remaining Ca was significant, resulting in high molar ratios, for example, 269 in favour of Mg when EGTA was used, proving that the micro-additions of selective coordination agents can potentially significantly alter the final results at the expense of only small additions of these reagents. While the non-chelating route already provides a Mg-product with low traces of Ca, the chelating route may be advantageous when a particularly low residual Ca content is required in the end product for certain applications.

A sample of Slovenian dolomite deposits labelled DOL1, identified as the most appropriate model of dolomite due to its low impurity content, favourable Ca:Mg molar ratio, and availability, was used to obtain these results. This provides a template to explore the potential of other Slovenian dolomites that contain other metal impurities that could affect Mg extraction. Further work should thus include the testing and validation of the developed procedures to potentially change or optimise the conditions to account for the natural variability in dolomite samples.

The end products Mg(OH)_2_ and MgO are important magnesium compounds with a variety of uses in many industries. These products could also be used as feedstock material instead of dolomite in metallic Mg production by thermal reduction procedures, replacing the calcination step and simultaneously removing the Ca and other potential impurities in dolomite that could affect the reduction step. The CaSO_4_ by-product from the first step of this procedure could be a valuable resource as well, either as the compound itself or for the production of metallic Ca.

As this procedure was developed on a laboratory-scale basis, the leap to commercial and industrial production needs further work and research into the underlying processes governing the dissolution of dolomite with H_2_SO_4_, the precipitation of Mg(OH)_2_, and other operational steps. Scaling up to an industrial level would also benefit from lifecycle assessments (LCAs) against traditional Pidgeon processes or seawater extraction routes.

## Figures and Tables

**Table 1:** Dolomite rock samples selected for this study—their labels, geological period, and appearance (colour).

Dolomite Sample	Geological Period	Appearance (Colour)
DOL1	Early to Middle Triassic	white
DOL13	Late Jurassic	light grey
DOL27	Late Triassic	dull white
DOL37	Permian	dull white
DOL43	Cretaceous	light grey

**Table 2:** Main elemental composition of the dolomite samples and major impurities (>100 ppm).

Dolomite Sample	Ca [wt.%]	Mg [wt.%]	Ca:Mg Molar Ratio [1:x]	Major Impurities [ppm]
DOL1	21.94 ± 0.13	12.750 ± 0.055	0.959	Zn (350), Fe (142), Mn (121), Si (110)
DOL13	22.29 ± 0.14	12.998 ± 0.085	0.962	Si (1900), Al (310), K (238), Na (222), Fe (203)
DOL27	22.30 ± 0.21	12.82 ± 0.17	0.948	Si (1100), Al (650), Fe (579), K (427), Na (131)
DOL37	22.203 ± 0.040	13.001 ± 0.023	0.966	Si (4000), Fe (730), Al (700), K (215), Na (191)
DOL43	24.05 ± 0.10	11.690 ± 0.086	0.802	Si (700), Na (171), Sr (117), S (105)

**Table 3:** Results of selective leaching of DOL1 with H_2_SO_4_. Results given as elemental yield *η* and Ca:Mg molar ratio of the resulting solution.

Experiment	*η*(Ca) [%]	*η*(Mg) [%]	Ca:Mg Molar Ratio [1:x]
DOL1-S-50-10 ^1^	5.49 ± 0.07	48.1 ± 0.4	8.39
DOL1-S-50-20	7.25 ± 0.14	47.8 ± 0.5	6.32
DOL1-S-105-5	4.89 ± 0.67	86.7 ± 2.6	17.0
DOL1-S-105-7.5	5.38 ± 0.72	97.9 ± 2.2	17.5
DOL1-S-105-10	4.81 ± 0.09	95.6 ± 1.5	19.1
DOL1-S-105-20	6.73 ± 0.14	94.9 ± 1.4	13.5
DOL1-S-150-10	5.60 ± 0.57	93.5 ± 0.7	16.0
DOL1-S-150-20	7.60 ± 0.14	95.0 ± 1.3	12.0

^1^ labelled as dolomite sample—reagent (S = sulphuric(VI) acid)—stoichiometric amount of combined Mg and Ca in %—volume of leaching solution in mL.

**Table 4:** Results of selective precipitation of DOL1 dissolved in HNO_3_ solution with NaOH. Results given as elemental yield *η* and Ca:Mg molar ratio of the resulting precipitate.

Experiment	*η*(Ca) [%]	*η*(Mg) [%]	Ca:Mg Molar Ratio [1:x]
DOL1-NOH-105-10 ^1^	6.14 ± 0.52	90.8 ± 0.6	14.2
DOL1-NOH-105-20	5.7 ± 1.0	91.7 ± 1.2	15.4

^1^ labelled as dolomite sample—reagents (N = nitric(V) acid, OH = NaOH)—stoichiometric amount of Mg in %—volume of NaOH solution in mL.

**Table 5:** ICP-OES elemental analysis results from different phases of the combined two-step procedure. Results given as elemental yield *η* and Ca:Mg molar ratio in the phase.

Phase	*η*(Ca) [%]	*η*(Mg) [%]	Ca:Mg Molar Ratio [1:x]
MgSO_4 (aq)_	3.57 ± 0.12	96.7 ± 1.2	26.0
CaSO_4 (s)_	92.4 ± 1.1	0.86 ± 0.20	0.009
Mg(OH)_2 (s)_	0.94 ± 0.13	94.8 ± 5.4	96.7
solution after precipitation	2.34 ± 0.21	0.38 ± 0.05	0.158
MgO _(s)_	0.92 ± 0.13	95.8 ± 4.6	99.6

**Table 6:** ICP-OES elemental analysis results from final phases of the optimised two-step procedure. Results given as elemental yield *η* and Ca:Mg molar ratio in the phase.

Chelating Agent	Phase	*η*(Ca) [%]	*η*(Mg) [%]	Ca:Mg Molar Ratio [1:x]
EDTA	Mg(OH)_2 (s)_	0.40 ± 0.12	91.4 ± 4.2	221
	solution after precipitation	2.85 ± 0.21	0.85 ± 0.35	0.286
EGTA	Mg(OH)_2 (s)_	0.33 ± 0.09	91.4 ± 2.1	269
	solution after precipitation	3.03 ± 0.16	0.65 ± 0.17	0.204
DTPA	Mg(OH)_2 (s)_	0.43 ± 0.07	92.0 ± 3.1	206
	solution after precipitation	2.83 ± 0.42	1.72 ± 0.55	0.584

## Data Availability

The original contributions presented in this study are included in the article/Supplementary Materials. Further inquiries can be directed to the corresponding author.

## Supplementary Materials

The following supporting information can be downloaded at: https://www.mdpi.com/article/10.3390/ma19204300/s1, Figure S1: (a) SEM image and (b) EDS analysis and results of the marked area of DOL1; Figure S2: TGA curves of DOL1 in air and argon; Figure S3: EDS analysis and results of the marked area of (a) CaSO_4_, (b) Mg(OH)_2_, and (c) MgO; Figure S4: XRD analysis of CaSO_4_; Figure S5: XRD analysis of Mg(OH)_2_; Table S1: List of all impurities in the dolomite samples; Table S2: Formation constants of Mg and Ca with EDTA, EGTA, and DTPA (at *I* = 0 mol/L and *T* = 25 °C).

## Abbreviations

The following abbreviations are used in this manuscript:

DTPA	Diethylenetriaminepentaacetic acid
EDS	Energy-dispersive X-ray spectroscopy
EDTA	Ethylenediaminetetraacetic acid
EGTA	Ethylene glycol-bis(β-aminoethyl ether)-N,N,N′,N′-tetraacetic acid
EU	European Union
ICP-OES	Inductively coupled plasma optical emission spectrometry
K_sp_	Solubility product
LOI	Loss on ignition
PTFE	Polytetrafluoroethylene
SEM	Scanning electron microscopy
TGA	Thermogravimetric analysis
wt.%	Mass percentage
XRD	X-ray diffraction
