# Supplementary material for: Extraction of Various Magnesium Species from Slovenian Dolomites Using Chemical and Hydrometallurgical Approaches
Source: Materials (Basel). 2026 Oct 13;19(20):4300. doi: 10.3390/ma19204300 (PMC13706612; doi:10.3390/ma19204300)
Supplement: Supplementary file 1 [file materials-19-04300-s001.zip › materials-4575905-supplementary.pdf]

Table S1. List of all impurities in the dolomite samples.

DOL1		DOL13		DOL27		DOL37		DOL43	
Element	*w* [ppm]	Element	*w* [ppm]	Element	*w* [ppm]	Element	*w* [ppm]	Element	*w* [ppm]
Zn	350 ± 10	Si	1900 ± 300	Si	1100 ± 140	Si	4000 ± 500	Si	700 ± 200
Fe	142 ± 2	Al	310 ± 30	Al	650 ± 20	Fe	730 ± 30	Na	175 ± 4
Mn	121.5 ± 0.7	K	238 ± 3	Fe	579 ± 4	Al	700 ± 4	Sr	117 ± 1
Si	110 ± 7	Na	222 ± 3	K	427 ± 3	K	215 ± 3	S	105 ± 8
Al	83 ± 5	Fe	203 ± 3	Na	131 ± 1	Na	190.9 ± 0.6	Al	93.0 ± 0.5
Na	64 ± 2	S	80 ± 20	P	69 ± 5	Sr	70.9 ± 0.2	Fe	48 ± 5
Pb	27 ± 2	Sr	73.7 ± 0.8	Sr	68.9 ± 0.6	Mn	44.3 ± 0.3	K	35.8 ± 0.5
Sr	26.80 ± 0.03	P	22 ± 4	Mn	30.2 ± 0.7	Ti	25.5 ± 0.5	P	17 ± 3
P	25 ± 2	Mn	18.7 ± 0.1	Pb	23 ± 2	P	18 ± 4	V	5.8 ± 0.3
K	9.5 ± 0.8	Ti	17.2 ± 0.5	Ti	22.1 ± 0.4	Zn	7.2 ± 0.4	Mn	3.38 ± 0.04
Ba	3.7 ± 0.7	V	5.1 ± 0.2	Zn	6.4 ± 0.2	V	6.9 ± 0.3	Cr	1.6 ± 0.2
Cd	2.9 ± 0.2	Cr	2.3 ± 0.3	V	3.6 ± 0.2	Ba	2.53 ± 0.01		
Cu	1.4 ± 0.8	Ba	1.54 ± 0.01	Cr	2.73 ± 0.05	Ni	2.1 ± 0.2		
Co	1.2 ± 0.2	Li	1.44 ± 0.02	Ba	2.33 ± 0.03	Cr	1.5 ± 0.1		
Ti	1.15 ± 0.07	Zn	1.3 ± 0.2	Li	1.61 ± 0.02	Li	1.25 ± 0.02		

Figure S1. (a) SEM image and (b) EDS analysis and results of the marked area of DOL1.

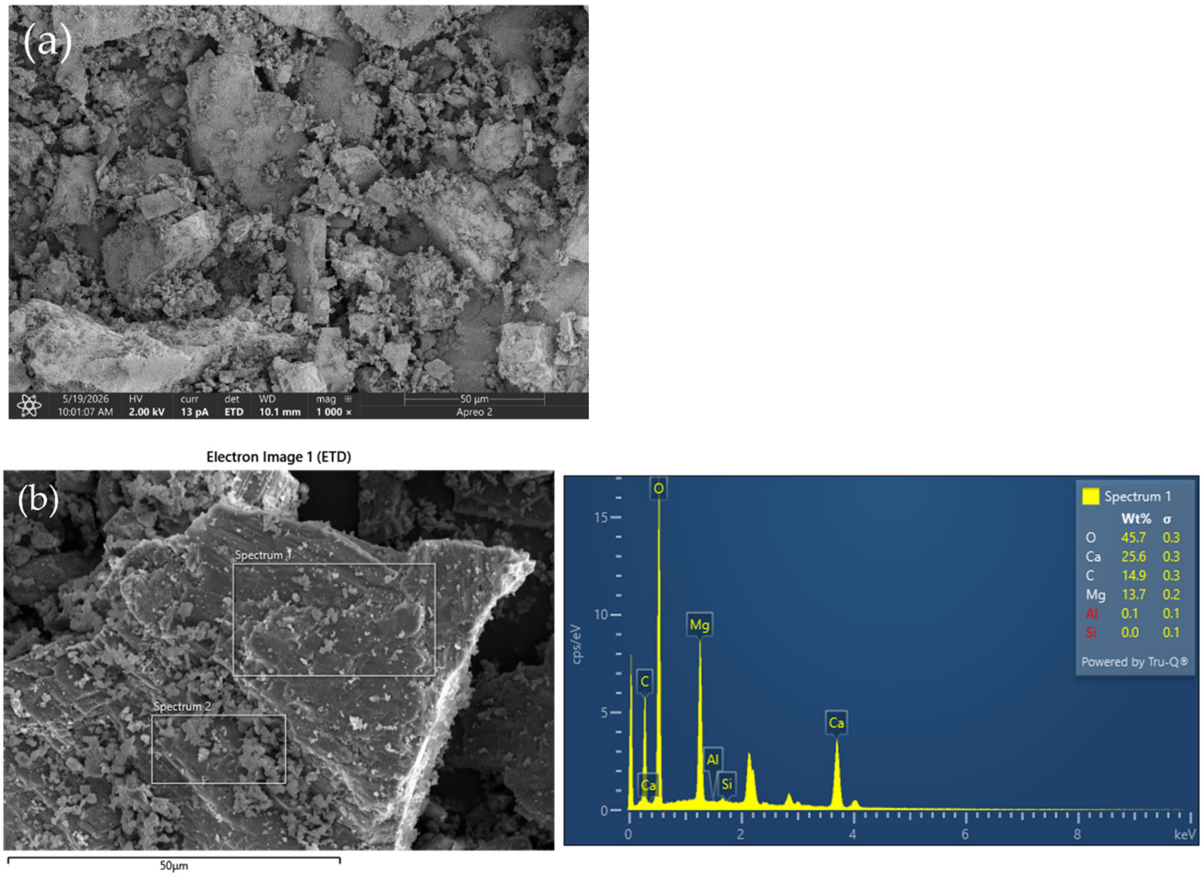

Figure S2. TGA curves of DOL1 in air and argon.

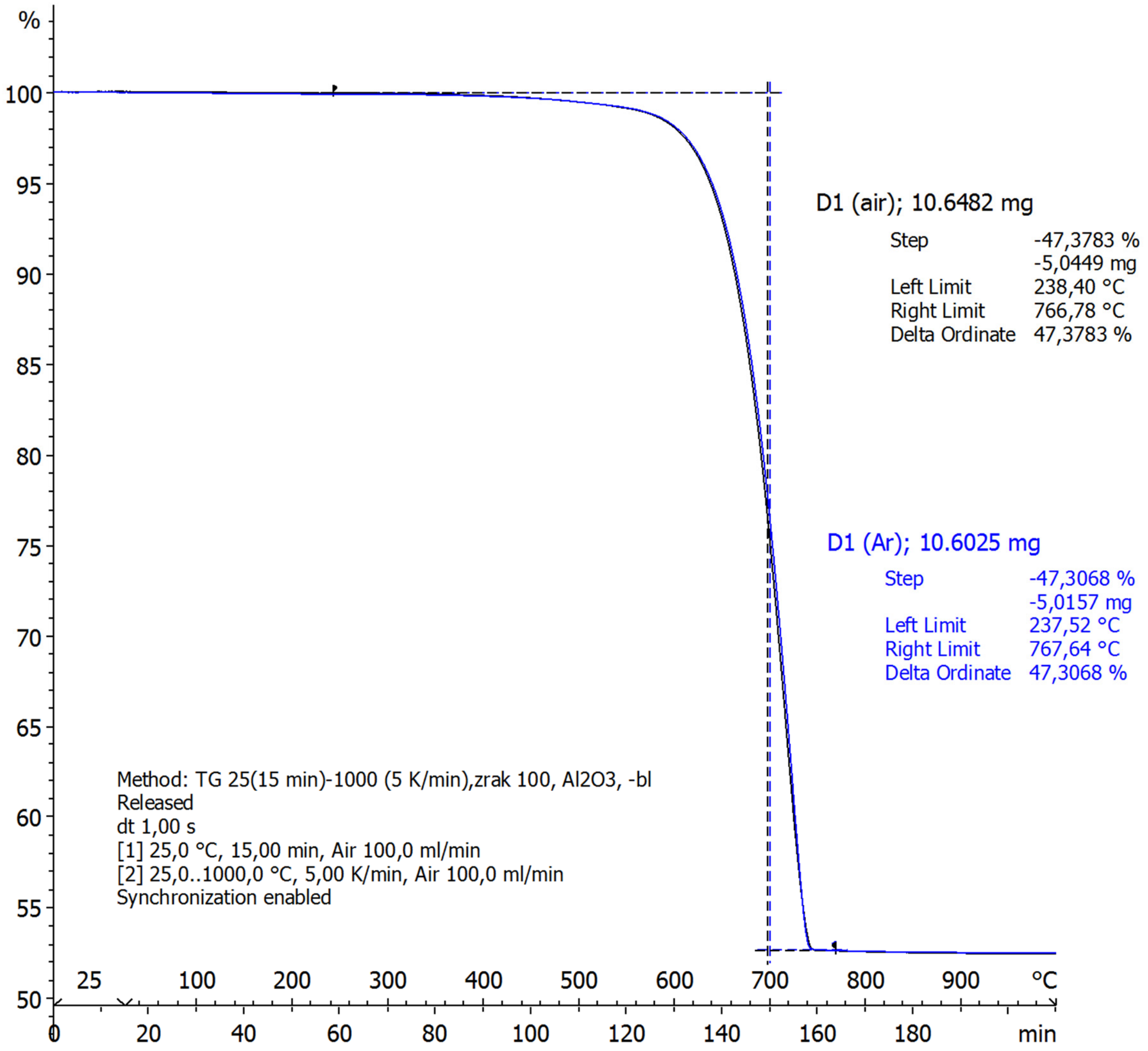

Figure S3. EDS analysis and results of the marked area of (a) $CaSO_4$, (b) $Mg(OH)_2$, and (c) MgO.

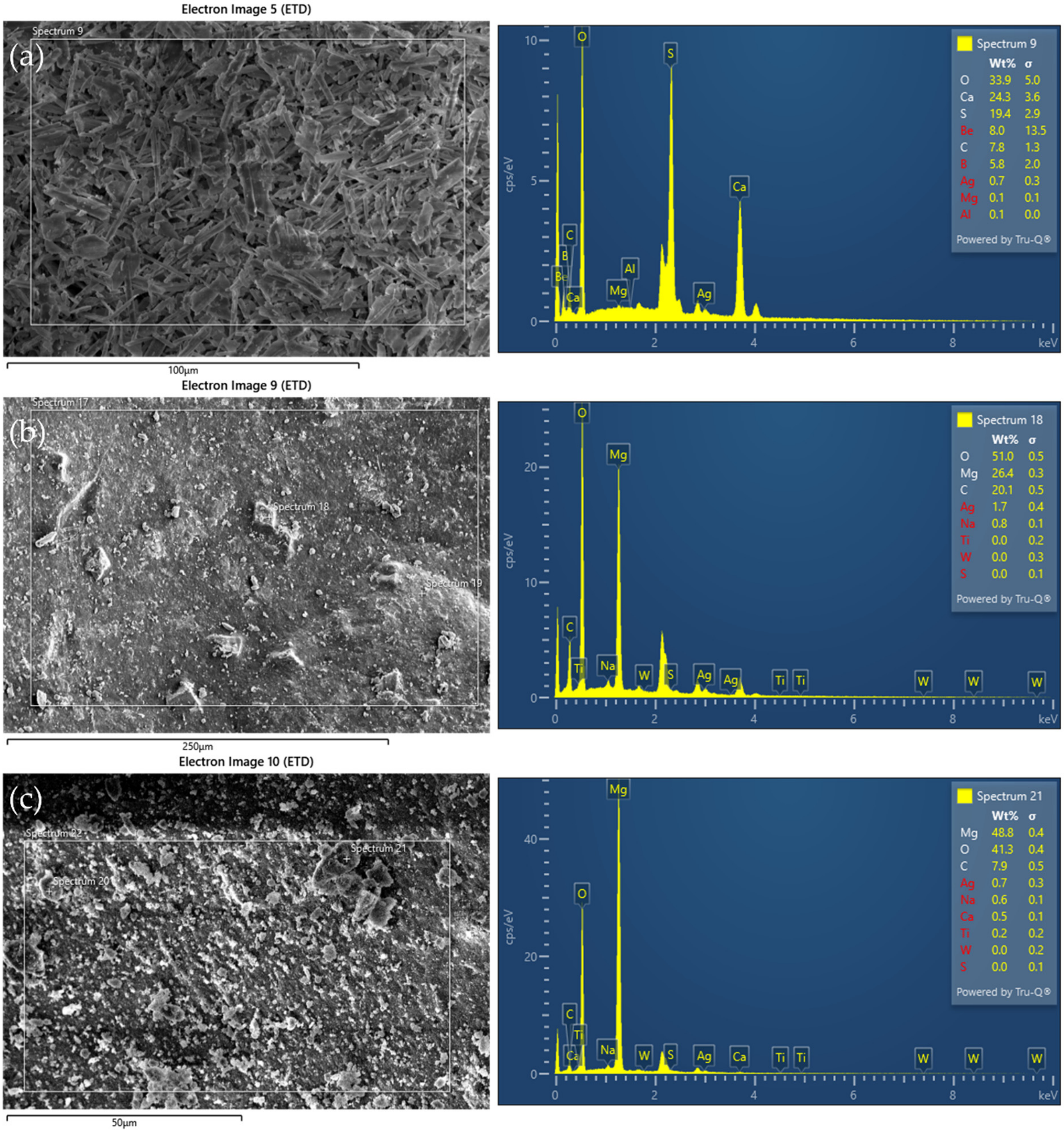

Figure S4. XRD analysis of $CaSO_4$.

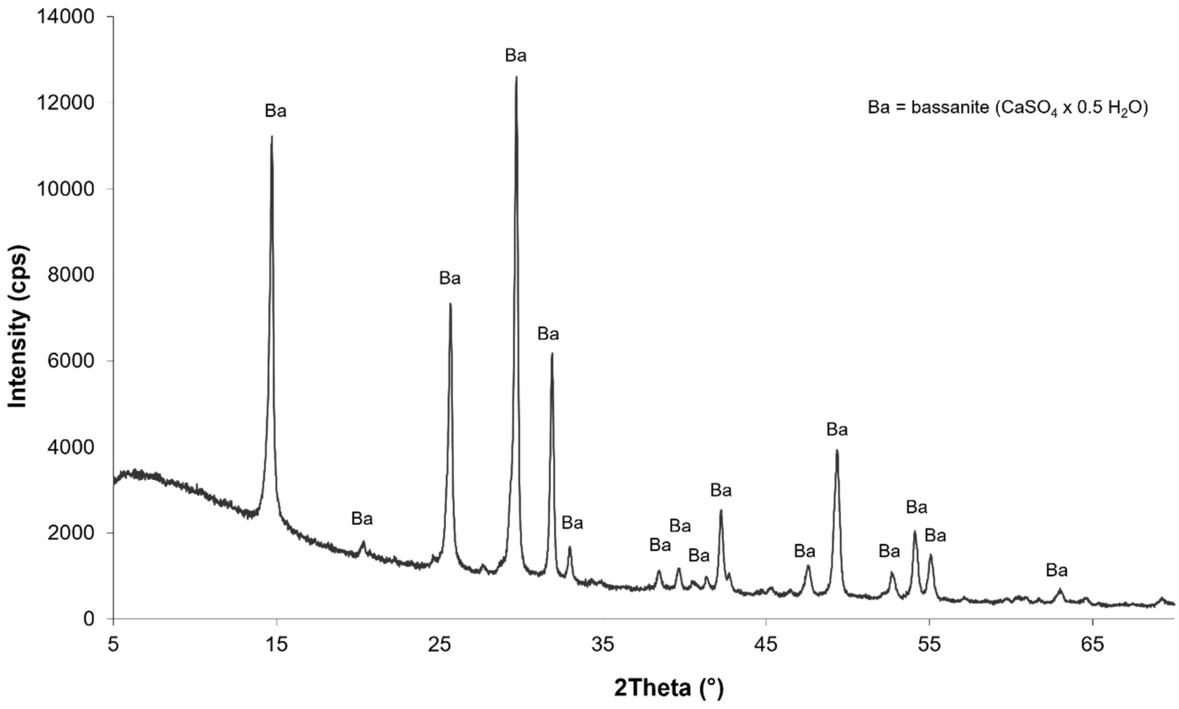

Figure S5. XRD analysis of $Mg(OH)_2$.

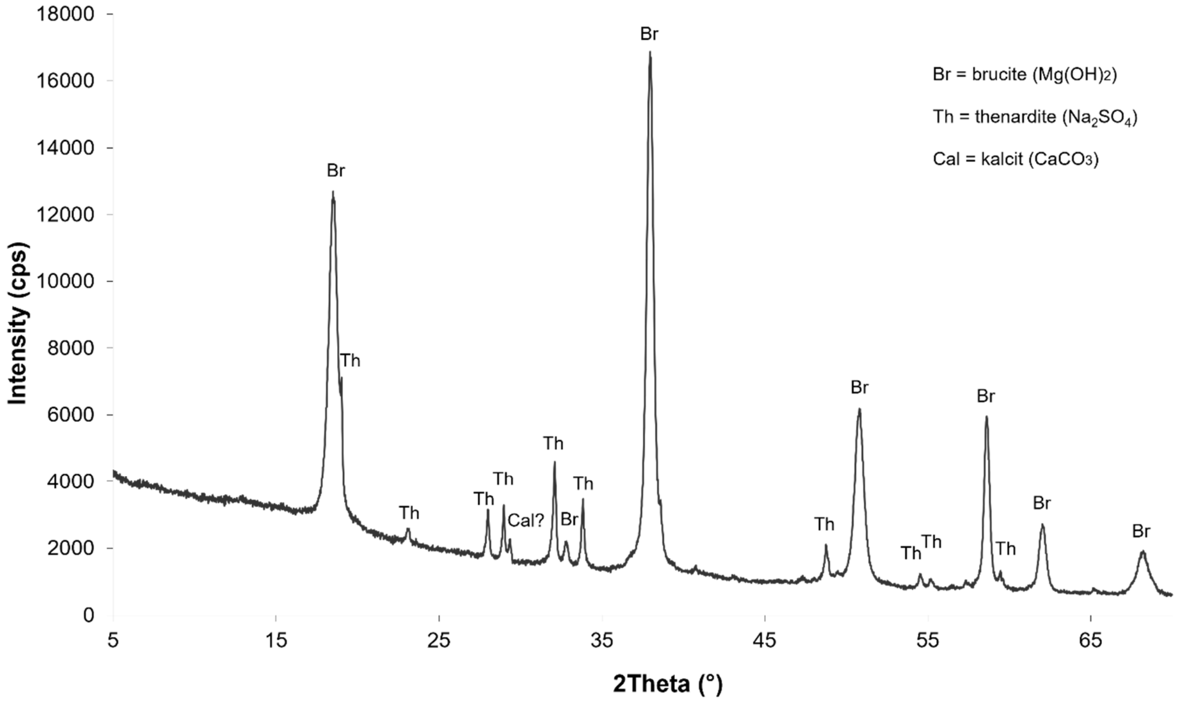

Table S2. Formation constants of Mg and Ca with EDTA, EGTA, and DTPA (at I = 0 mol/L and T = 25 °C). [55]

Chelating agent	$pK_f(Mg)$	$pK_f(Ca)$
EDTA	10.70	12.57
EGTA	7.08	12.81
DTPA	11.27	12.76
